# Diagnostic value of the biochemical tests in patients with purulent pericarditis

**DOI:** 10.12669/pjms.304.3743

**Published:** 2014

**Authors:** Meral Ekim, Hasan Ekim

**Affiliations:** 1 Meral Ekim, MD, Department of Biochemistry, Bozok University School of Medicine, Yozgat, Turkey.; 2 Hasan Ekim, MD, Department of Cardiovascular Surgery, Bozok University School of Medicine, Yozgat, Turkey.

**Keywords:** Pericarditis, Purulent Effusion, Biochemical Tests

## Abstract

***Objectives:*** Purulent pericarditis is a collection of purulent effusion in the pericardial space. It has become a rare entity with the increased availability and use of antibiotics. In contrast to pleural empyema, there are few data regarding the biochemical parameters of purulent pericardial effusion to aid diagnosis. Therefore, in this study, we have evaluated the diagnostic utility of biochemical tests in patients with purulent pericarditis.

***Methods:*** Between September 2004 and September 2012, we treated fifteen children with purulent pericarditis and tamponade. There were 8 boys and 7 girls, ranging in age from 8 months to 14 years, with a mean age of 5.3 ± 3.2 years. Echocardiographic diagnosis of cardiac tamponade was made in all patients. All patients underwent immediate surgical drainage due to cardiac tamponade. The diagnosis of purulent pericarditis was supported by biochemical tests. Anterior mini-thoracotomy or subxiphoid approach was performed for surgical drainage.

***Results:*** The most common clinical findings were tamponade, hepatomegaly, tachycardia, fever refractory antibiotic therapy, dyspnea, tachypnea, cough, and increased jugular venous pressure. Central venous pressure decreased and arterial tension increased immediately after the evacuation of purulent effusion during operation in all patients. The pericardial effusion had high lactic dehydrogenase, and low glucose concentration, confirming purulent pericarditis. Also, pH (mean± SD) was 7.01 ± 0.06. The culture of pericardial effusions and blood samples were negative.

***Conclusion:*** Biochemical tests are useful guideline when assessing the pericardial effusions. However, these tests should be interpreted with the clinical and operative findings.

## INTRODUCTION

Purulent pericarditis is a collection of purulent effusion in the pericardial space. It has become a rare entity with the increased availability and use of antibiotics. Diagnosis can be challenging especially when antimicrobials have been initiated before appropriate culture specimens are taken.^1^ Clinical diagnosis is often delayed until severe symptoms and signs of cardiac tamponade arise.^2^ Unfortunately, the diagnosis is most often made at autopsy, and mortality remains high in diagnosed cases despite aggressive drainage and prolonged antibiotic therapy.^3^

Purulent pericarditis is a rare entity in the developed countries. It generally presents with acute cardiovascular decompensation and a sepsis-like appearance.^4^ The postulated pathophysiology has been that the adjacent pleuropulmonary infection may cause an inflammatory response in the pericardium with migration of neutrophyls and eventual deposition of fibrin.^5^ Since the pericardial space is only rarely the initial site of infection, identification of the primary focus is mandatory. Hematogenous septic dissemination, direct spread via infected tissue into the neighborhood, or local complication following cardiac surgery is most common routes of infection.^5^ It is most often a result of the spread of a contiguous pulmonary, intracardiac, or chest wall infection.^6^

In contrast to pleural empyema, there are few data regarding the biochemical parameters of purulent pericardial effusion to aid diagnosis. The main reason behind the scarcity of biochemical analyzing of pericardial effusion is the difficulty of collecting pericardial effusion samples. Therefore, in this study, we have evaluated the diagnostic utility of biochemical tests in patients with purulent pericarditis.

## METHODS

Between September 2004 and September 2012, fifteen children with purulent pericarditis and tamponade were treated in Dursun Odabas Mediacal Center, Van, Turkey. There were 8 boys and 7 girls, ranging in age from 8 months to 14 years, with a mean age of 5.3±3.2 years. All patients were transferred from local hospitals.

Serial electrocardiograms, chest radiographs, echocardiograms, and hemoglobin measurements were done in all patients, and samples of blood and pericardial fluid were taken for culture, histological and biochemical analyses as part of the routine diagnosis and were later used to conduct this study. Echocardiographic diagnosis of cardiac tamponade was made in all patients. To examine the diagnostic utility of pericardial lactate dehydrogenase (LDH), pericardial glucose level and several other biochemical parameters in purulent pericarditis, all aspirates were collected in tubes without anticoagulant and preserved at suitable conditions. Then these samples were sent to the laboratory where they were centrifuged. But, samples for pH testing were collected in a heparinised syringe and tested immediately using a blood gas analyzer.

Three patients without loculation or fibrotic changes underwent echo-guided pericardiocentesis before surgery. However, pericardiocentesis yielded very little purulent aspirate due to thick effusion or multiple loculations. All patients underwent immediate surgical drainage due to cardiac tamponade. The diagnosis of purulent pericarditis was supported by biochemical tests, such as pH, lactic dehydrogenase, protein, and glucose concentrations of the pericardial effusion in all patients.

The initiation of mechanical ventilation in a patient with tamponade may produce a sudden drop in blood pressure because the positive intrathoracic pressure will contribute to further impairment of cardiac filling. Therefore, subxiphoid approach using local anesthesia was preferred in these patients without loculations. The remaining twelve patients who underwent general anesthesia were stained and draped before endotracheal intubation to avoid impending cardiac arrest. 

Anterior mini-thoracotomy or subxiphoid approach was performed for surgical drainage. At thoracotomy, a left anterior mini-thoracotomy was performed between 4^th^ and 5^th^ intercostals spaces. A portion of the pericardium was resected 4 cm in diameter. The pericardial effusion was aspirated. Then, loculations were unified with finger. Then, pericardial cavity was washed and two chest tubes were inserted into the pericardial and pleural cavities, respectively. In 3 patients who had subxiphoid drainage, a portion of the pericardium was resected and effusion was aspirated. Postoperatively, fibrinolytic therapy was performed with streptokinase through the chest tube in these 3 patients. Streptokinase interacts with plasminogen to convert plasminogen to plasmin, which dissolves the fibrinous components. Streptokinase (15000 units/kg) was dissolved in 50 ml of isotonic solution and warmed to body temperature to prevent arrhythmias.^7^ The chest tube was clamped and streptokinase was then instillated into the pericardial space and the chest tube remained clamped for two hours after instillation. Instillation was performed twice daily until the drainage had ceased.

## RESULTS

The most common clinical findings were tamponade, hepatomegaly, tachycardia, fever refractory antibiotic therapy, dyspnea, tachypnea, cough, and increased jugular venous pressure.

Electrocardiography showed decreased voltage. Chest radiographs revealed cardiopericardial shadow enlargement in all patients and left pleural effusion in four patients. Echocardiography showed massive pericardial effusion and right atrial collapse during early systole in all patients. 

Central venous pressure decreased and arterial tension increased immediately after the evacuation purulent effusion during operation in all patients. Chest tubes remained in place until the drainage fluid became clear and minimal in quantity with no further accumulation of effusion around the heart as assessed by transthoracic echocardiography. The average volume of pericardial fluid drained was 315±29 (140-900) ml. The macroscopic appearance of the pericardial fluid was serofibrinous in 8 patient, and purulent in 7 patients. There was concurrent trapped lung in three patients. The culture of pericardial effusions and blood samples were negative. Histopathological examinations showed nonspecific inflammation of the pericardium in all patients. However, Biochemical analysis was remarkable for pericardial effusion LDH values that were greater than 1000 IU/L in all samples. Results of biochemical analyses in pericardial effusion samples are shown in table 1. The samples of pericardial effusion had high lactic dehydrogenase and protein concentrations, but low glucose concentrations. The mean LDH, protein and glucose levels were 3128±42 IU/L, 22±1.2 g/dl and 28.5±2.3 mg/dl, respectively. Also, the mean pH was 7.01 ± 0.06. Results of all biochemical parameters were in favor of purulent pericarditis.

There were no intraoperative or postoperative complications. Postoperative ventilation was not required for any patient. Oral intake and ambulation of the patients were resumed on the second postoperative day. Postoperative echocardiography revealed normal size of the heart. No cardiac abnormalities were observed in any patient at the time of discharge.

## DISCUSSION

The pericardial space normally contains 15-50 ml of fluid, which serves as lubrication for the both pericardial layers. This fluid is thought to produce by the visceral pericardium and is essentially an ultrafiltrate of plasma. However, this fluid does not represent simply a lubricator between the pericardial sheets, but an important reservoir of mediators which may modulate cardiac cell functions.^8^

**Table-I:** Mean values of biochemical parameters of pericardial effusions

***Biochemical parameters ***	***(Mean ±SD )***
LDH	3128±42 IU/L
Glucose	28.5±2.3 mg/dl
Protein	22±1.2 g/dl
pH	7.01±0.06

Pericardial fluid is increased in a variety of pathologic conditions, including infectious, malignant, auto-immune and metabolic diseases.^9^ Transudative fluids result from obstruction of fluid drainage; exudative fluids occur secondary to inflammatory, infectious, malignant or auto-immune processes within the pericardium.^10^

Purulent pericarditis is also defined as a neutrophilic pericardial effusion infected by a bacterial, fungal, or parasitic agent. It may lead to tamponade and septic shock.^11^ The diagnosis of purulent pericarditis is based on high clinical suspicion from an accurate history and a through physical examination and is confirmed with a positive stain or culture of pericardial effusion or pericardium.^12^ Gram’s stain and cultures of pericardial effusions usually reveal the causative microorganism. Sometimes, initial empiric antibiotic therapy started by the referring clinicians occasionally prevents the production of the microorganism in pericardial effusion. Possibly, we failed to cultivate the causative agent due to previous antibiotic therapy in this series.

Purulent pericarditis is an acute severe illness with still high mortality of up to 30%, especially if diagnosis and treatment are delayed.^5^ In patients treated only with antibiotics without pericardial drainage, the rapid unsuspected development of a large pericardial effusion may result in sudden cardiovascular collapse due to cardiac tamponade^3^, as seen in this series. Various effective drainage techniques for purulent pericardial effusion are available. Purulent effusions can be removed with echo-guided pericardiocentesis, or subxiphoid catheter drainage with streptokinase. However, pericardiocentesis or catheter drainage may be inadequate due to thick effusion or multiple loculations. In these circumstances, subxiphoid pericardial window, mini-anterior thoracotomy and pleuropericardial window, video-assisted thoracoscopic approach may be performed. In this series, video-assisted thoracoscopy was not used to avoid pneumopericardium. Regardless of the chosen method, purulent pericarditis requires a timely and aggressive approach including antibiotics and prompt drainage of the purulent pericardial effusion.^13^

Purulent pericarditis shares pathophysiological similarities with empyema thoracis.^12^ The pathophysiology of empyema thoracis evolves through three distinct phases: the exudative phase, the fibrinopurulent stage, and the organizational stage.^14^

Experimental models argue for a similar pathophysiological process in purulent pericarditis, leading from pericardial inflammation to pericardial adhesions and fibrosis.^15^^,^^16^ Therefore, we estimate that similar changes occur in the pericardial cavity when the process occurs in the pericardium.

The biochemical analysis of pericardial effusion is a good diagnostic tool in the appropriate management of the most probable etiologies of the effusion. Combination of different tests tended to perform better and demonstrated higher sensitivities and odds ratios compared with individual tests.^17^

Ideally, determining biochemical parameters for pericardial effusion is best performed during the first hour after sample collection. However, it is important to consider that pericardial effusion samples are obtained in an emergency situation perhaps based on patient’s clinical condition. If the samples are taken during night or weekend, as was done in this series, requested biochemical tests are not available when they are ordered. Therefore, samples should be preserved at suitable temperature. Antonangelo et al.^18^ observed that with the exception of glucose, the results obtained for protein and LDH did not vary significantly for the tests performed in the pleural fluid samples maintained at room temperature up to day 4 of the study. Their samples maintained under refrigeration remained stable for the first 7 days of the study regarding protein and glucose. But, samples for pH testing should be collected in a heparinised syringe and tested immediately using a blood gas analyzer.^19^ We estimate that samples of pericardial fluid may be preserved similar conditions (same temperature and storage time). 

In contrast to the relatively well-documented ability of biochemical tests on pleural and peritoneal fluids, few studies have analyzed the differentiation of pericardial fluids into transudates and exudates. A study by Meyers et al.^20^ demonstrated that exudates are best described by the following biochemical parameters: specific gravity>1.015, pericardial fluid protein level >30 g/l, and pericardial fluid LDH level>300 U/l. The purulent pericardial fluid glucose levels vary; glucose concentrations less than 35 mg/dl have been described.^10^ A further case series noted that inflammatory pericardial effusion had a mean ± SD PH of 7.06±0.07 compared with non-inflammatory effusion (7.42±0.06).^20^ In this series, mean pH ± SD was 7.01±0.06 suggesting purulent effusion. The more likely increased effusion LDH is due to probable preferential leak from the adjacent perimyocardial tissue. The increased protein level may be more difficult to explain.^15^

In the fibrinopurulent stage, the pleural effusion is characterized by positive bacterial studies, a glucose level below 60 mg/dl, a pH below 7.20, and a pleural effusion LDH more than three times the upper limit for serum.^21^ We believe that Right's criteria are applicable to pericardial effusions. Therefore, these biochemical parameters may be used in the assessing patients with pericardial effusion. In our series, all patients have met these criteria except positive bacterial study.

The fibrin accumulation caused by the purulent effusion preventing effective drainage can be cleared by the fibrinolytic effect of streptokinase.^9^ We estimate that streptokinase plus DNase may be useful in purulent Pericarditis. However, in patients with dense loculations and fibrotic changes, catheter drainage and irrigation with thromboliytic agents may not prevent cardiac tamponade and persistent purulent effusion. In this situation, partial pericardial resection or pericardiectomy seems to be associated with a better outcome than simple catheter drainage.^11^

Our findings suggest that biochemical tests such as pH, protein, glucose, and LDH levels should be considered to serve a valuable adjunctive role in assessing patients with purulent pericarditis. Of these test components of the proposed modification of the Light criteria, the fluid LDH level has the highest discriminatory power^17^, as seen in this series.

## CONCLUSION

In patients with purulent pericarditis, early surgical drainage combined with antibiotic therapy should be performed to prevent loculations and fibrotic changes. Sometimes, identification of causative bacterial agents might have failed due to previous antibiotic therapy. In this situation, biochemical tests are useful guideline when assessing the pericardial effusions. However, these tests should be interpreted with the clinical and operative findings.
